# Progress and outcomes of health systems reform in the United Arab Emirates: a systematic review

**DOI:** 10.1186/s12913-017-2597-1

**Published:** 2017-09-20

**Authors:** Erik Koornneef, Paul Robben, Iain Blair

**Affiliations:** 1 Institute for Health Policy and Management, Erasmus University, Rotterdam, The Netherlands; 2 Truven Health Analytics, An IBM Company, Michigan, USA; 3 Dutch Healthcare Inspectorate, Utrecht, The Netherlands; 4 Institute of Public Health, College of Medicine and Health Sciences, United Arab Emirates University, PO Box 17666, Al Ain, United Arab Emirates

**Keywords:** Health system, Reform, Middle East, United Arab Emirates, Health insurance, Privatization

## Abstract

**Background:**

The United Arab Emirates (UAE) government aspires to build a world class health system to improve the quality of healthcare and the health outcomes for its population. To achieve this it has implemented extensive health system reforms in the past 10 years. The nature, extent and success of these reforms has not recently been comprehensively reviewed. In this paper we review the progress and outcomes of health systems reform in the UAE.

**Methods:**

We searched relevant databases and other sources to identify published and unpublished studies and other data available between 01 January 2002 and 31 March 2016. Eligible studies were appraised and data were descriptively and narratively synthesized.

**Results:**

Seventeen studies were included covering the following themes: the UAE health system, population health, the burden of disease, healthcare financing, healthcare workforce and the impact of reforms. Few, if any, studies prospectively set out to define and measure outcomes. A central part of the reforms has been the introduction of mandatory private health insurance, the development of the private sector and the separation of planning and regulatory responsibilities from provider functions. The review confirmed the commitment of the UAE to build a world class health system but amongst researchers and commentators opinion is divided on whether the reforms have been successful although patient satisfaction with services appears high and there are some positive indications including increasing coverage of hospital accreditation. The UAE has a rapidly growing population with a unique age and sex distribution, there have been notable successes in improving child and maternal mortality and extending life expectancy but there are high levels of chronic diseases. The relevance of the reforms for public health and their impact on the determinants of chronic diseases have been questioned.

**Conclusions:**

From the existing research literature it is not possible to conclude whether UAE health system reforms are working. We recommend that research should continue in this area but that research questions should be more clearly defined, focusing whenever possible on outcomes rather than processes.

**Electronic supplementary material:**

The online version of this article (10.1186/s12913-017-2597-1) contains supplementary material, which is available to authorized users.

## Background

**Fig. 1:**
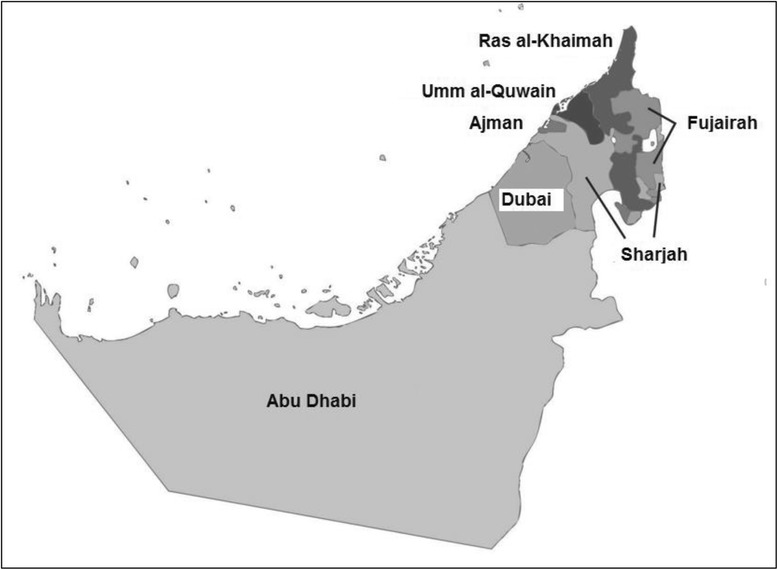
Map of United Arab Emirates showing the seven Emirates

The United Arab Emirates (UAE) is a young nation, established in 1971 as a federation of seven Emirates: Abu Dhabi, Dubai, Ajman, Umm Al Quwain, Sharjah, Fujairah and Ras Al Khaimah (Fig. 1). This newness has allowed its leaders to deliberately plan for the development of UAE society in order to strengthen national unity, promote continuous economic growth and personal health and wellbeing [1].

As recently as the late 1960s, in the UAE, it was reported that only half of newborn babies survived and one in three mothers died during childbirth [2]. Almost 50 years later many health outcomes are on par or even better than those seen in developed countries. The maternal mortality ratio (MMR) is now 8 per 100,000 live births (in contrast to an MMR of 14 in the USA) and the infant mortality rate is 5.6 per 1000 live births (5.8 in the USA) [3]. Healthcare in the UAE has benefited from rapid economic growth and there has been a significant increase in the number of healthcare facilities and healthcare professionals and in levels of service use. For example, between 2011 and 2015 healthcare spending in the UAE grew by 10% to US$ 11 billion [4].

In 2014, the Vice President and Prime Minister of the UAE, His Highness Sheikh Mohammed bin Rashid Al Maktoum, launched an ambitious set of plans with the overall goal of making the UAE one of the best countries in the world by 2021, the 50th anniversary of its foundation. The *UAE National Agenda 2021* consists of a comprehensive set of key performance indicators (KPI) with specific targets and clear pathways for achieving those targets [5]. For example, in 2016, the UAE Government announced the appointment of a Minister of Happiness whose task it is to ensure that the UAE is ranked among the top five countries in the world according to the World Happiness Report [6].

The improvement of the health of its citizens and the performance of the healthcare system form one of seven headings of the UAE national strategy. The KPIs include population health targets, such as increasing life expectancy and reducing tobacco consumption, as well as more structural and organizational targets, such as the regulatory requirement for all healthcare facilities to be externally accredited [5]. Overall, the UAE aims to be ranked amongst the top 20 countries in the world, according to the Legatum Prosperity Indicator. In 2015 the UAE was ranked 34th globally, an improvement from 37th place in 2014 [7].

Given its starting point, it is remarkable what has been achieved in the UAE in the last four decades. However since the early 2000s the UAE has been involved with an ambitious program of health system reforms to further improve health and health services and to address cost and quality challenges. These reforms have focused on the introduction of private health insurance and encouraging the growth of private health provision against a back-drop of rapid population growth and a rising prevalence of chronic disease and chronic disease risk factors including obesity, low levels of physical activity and diabetes [8].

The purpose of this paper is to describe the main healthcare challenges and public health issues in the UAE and review the progress and outcomes of health systems reform. This will be achieved by reviewing secondary data from peer-reviewed journal publications and reports of government agencies and related health organizations.

Even though the term *health system reform* is regularly used, it is rarely defined in any operational way [9]. In this paper we have defined health system reform as “sustained, purposeful change to improve the efficiency, equity and effectiveness of the health care sector” [10].

## Methods

Data for this review were obtained by means of a systematic search of the published literature using defined keywords, conducted according to Preferred Reporting Items for Systematic Reviews and Meta-Analyses (PRISMA) guidelines [11]. MEDLINE (accessed by PubMed), EMBASE and PsycINFO electronic databases were searched covering the period from 2002 to April 2016 using a combination of the following MESH terms, free-text words, and entry terms: UAE; United Arab Emirates; Dubai; Abu Dhabi; healthcare quality, access and evaluation; healthcare reform, health system reform, health sector reform. In addition, reference lists of published studies were searched manually for relevant articles. To minimize publication bias and improve the usefulness of our review we also conducted a thorough analysis of existing, publicly available “grey” literature by means of personal contact with senior officers at health authorities, government agencies and health sector organizations and a review of publications and reports from health policy centers, the healthcare business sector and key international sources. These sources included the World Health Organization (WHO) and its regional office for the Eastern Mediterranean (EMRO), the Organisation for Economic Co-operation and Development (OECD), the World Bank, and local sources such as the Health Authority Abu Dhabi (HAAD), Dubai Health Authority (DHA), Ministry of Health and the Federal Competitiveness and Statistics Authority. Finally, a small number of other sources were reviewed from local “think tanks” and consultancy and research firms. These included Ernst and Young, Colliers International, The Economist Intelligence Unit, US-UAE Business Council, Joint Commission International and Sheikh Saud bin Saqr Al Qasimi Foundation for Policy Research. Eligible studies were those that focused on the UAE health system. Excluded studies were those that focused on healthcare in the wider region, studies that were published before 2002, articles that were not available in English and duplicate studies or those that formed part of a larger study. Two reviewers (EK, IB) independently screened the titles and abstracts of identified studies and duplicates were removed. Studies considered eligible for full text screening were retrieved for full review. The reviewers independently assessed the papers for eligibility and quality, and then met to resolve any disagreements regarding eligibility and/or quality. The key features of the studies were summarized using a data extraction form that recorded first author name, year, study design, setting, theme and key findings. A descriptive and narrative synthesis of the studies was carried out.

## Results

**Fig. 2:**
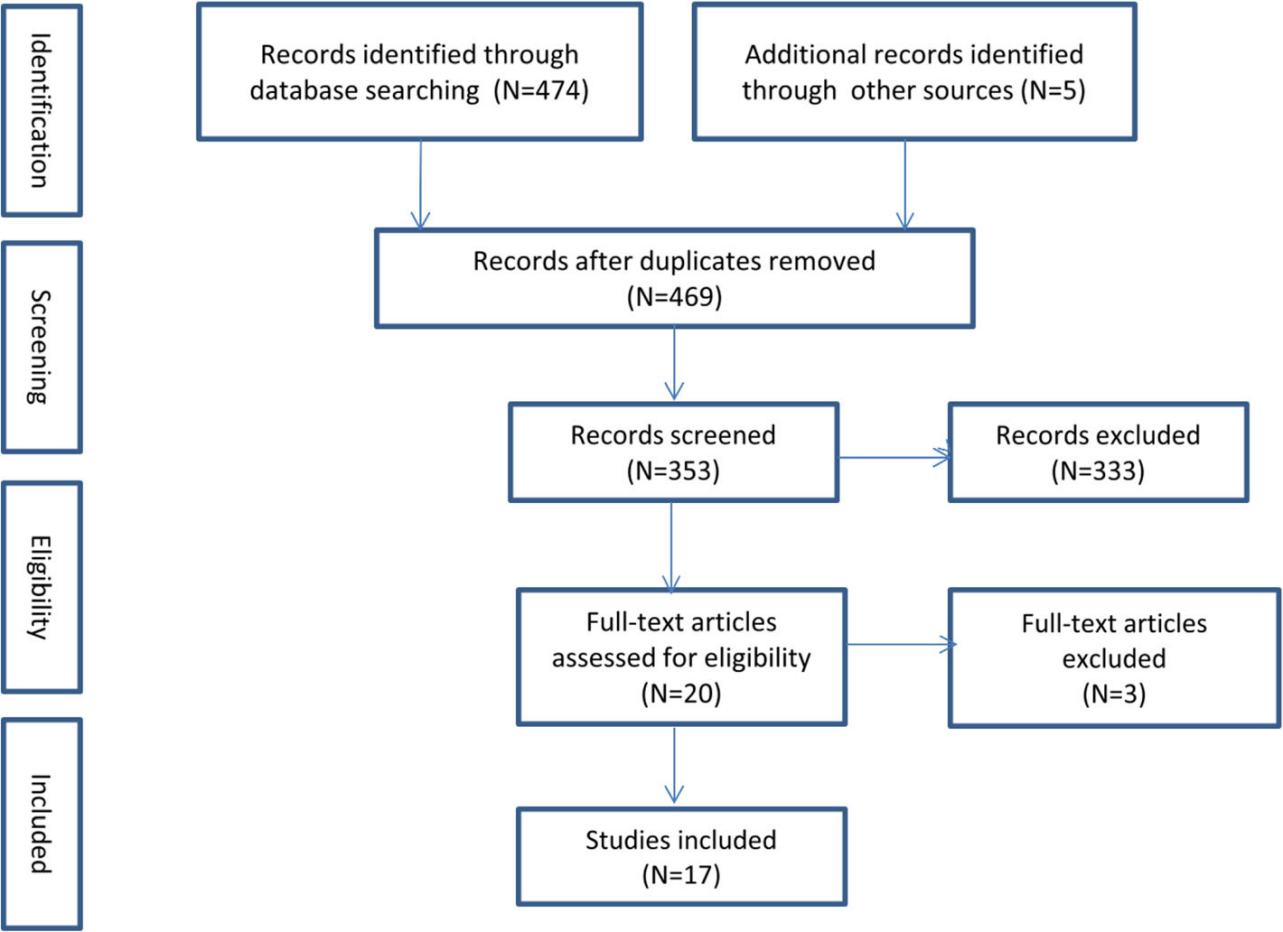
Flow diagram of the search and selection process

We screened 353 published articles and 17 met our inclusion criteria (Fig. 2). Of these, three related to Dubai, eight to Abu Dhabi and six were UAE-wide and all were published after 2010. There were four cross-sectional studies, six policy reviews, three data reviews, two case studies and two literature reviews. From a careful reading of the selected papers it was possible to classify the content into six categories or themes. The six themes are: the UAE health system, population health, the burden of disease, healthcare financing, healthcare infrastructure and workforce and the impact of reforms (Table 1). The findings are summarized under these headings in the following sections. For the sake of clarity, while acknowledging the possible inferior quality, we have included the grey literature, appropriately referenced, it in our summary along with the published literature.

**Table 1:** Summary of study characteristics included in the literature search

First author, Year	Study Design / Method	Focus	Topics	Key Findings
Al Maskari, 2010 [33]	Retrospective cohort study	Dubai	Healthcare Financing	Average costs (without complications): 1605 USD, with complications 5645 USD
			Burden of disease (diabetes)	61% of all diabetes patients in the cohort reported to have suffered poor health during the past month
Al Zaabi, 2014 [36]	Retrospective cohort study	Abu Dhabi	Healthcare Financing	Asthma treatment in the UAE costs around 200 USD per capita
			Burden of disease (asthma)	Crude prevalence of asthma is 4.8%, much lower than expected
Blair, 2012 [21]	Data review	UAE	Burden of disease	Substantial population growth
			Population Health	Data quality needs to be improved
Blair, 2012 [27]	Healthcare policy review	UAE	UAE Health System	Review of UAE healthcare system 2000–2010
			Population Health	Dramatic population growth, young population
			Burden of disease	Main causes of death: road injury, health and cerebrovascular diseases
			Healthcare Financing	Expenditure has grown from 1.7 billion USD in 2000 to 9.5 billion USD in 2011
			Healthcare infrastructure and workforce	Largely expat clinical workforce (>85%)
			Impact of reforms	Satisfaction appears high but citizens still opt for treatment abroad
Al Hosani, 2014 [31]	Healthcare policy review	UAE	Burden of disease	National neonatal screening program
Brownie, 2015 20]	Regulatory policy review	UAE	Healthcare infrastructure and workforce	Brief historical overview of regulation and licensing in the UAE
			UAE Health System	Move towards central, consistent regulation and licensure
Hajat, 2012 [34]	Retrospective cohort study	Abu Dhabi	Burden of disease	This population-wide cardiovascular screening program demonstrated a high cardiovascular burden for our small sample in Abu Dhabi
Hajat, 2012 [29]	Healthcare policy review	Abu Dhabi	Burden of disease	Largely unhealthy lifestyle - lack of physical activity, poor diets & tobacco consumption
			Population Health	Weqaya - a program aimed at improving population health (cardiovascular)
			Healthcare Financing	Diabetes may cost up to $1.1 billion per year in Abu Dhabi
			UAE Health System	Weqaya program - screened 94% of national population
Hamidi, 2014 [19]	Focused literature review	Abu Dhabi	Healthcare Financing	In Abu Dhabi there has been a significant growth in demand for healthcare since 2007
			UAE Health System	Strategies are in place designed to slow the rise in spending
Hamidi, 2015 [38]	Data review	Abu Dhabi	UAE Health System	The health care model has not fully matured yet and needs to focus on creating a sustainable model that is affordable and provides high quality, safe care
Hamidi, 2015 [17]	Data analysis	Dubai	UAE Health System	Changes required to move from curative to preventive care and from inpatient to day care, outpatient and home-based care
			Healthcare infrastructure and workforce	Cost containment for pharmaceuticals
			Healthcare Financing	Strengthen long-term care
Koornneef, 2012 [14]	Healthcare policy review	Abu Dhabi	Impact of reforms	Limited information available, some evidence of improved access and patient satisfaction
			UAE Health System	Three key characteristics: centralized regulatory system, mandatory insurance and competition
Loney, 2013 [25]	Literature search	UAE	Population Health	UAE has significantly invested resources into population-based control measures
			Burden of disease	Top four priorities: cardio, injury, cancer, respiratory diseases
Mosaad, 2014 [28]	Healthcare policy review	UAE	UAE Health System	Risk factors: ageing population, population growth, health risk factors
			Healthcare infrastructure and workforce	Lack of clinical staff, hospital beds and referral network
			Impact of reforms	Progress is “underway” in the UAE with a focus on quality, screening and competition. However, the focus is not on prevention
Osenenko km et al., 2015 [50]	Retrospective cohort study	UAE	Population Health	Greater understanding of the factors leading to high adherence to guidelines would be useful
			Impact of reforms	Compared to international benchmarks, the patients in Dubai received similar quality outcomes
Sharif, 2011 [44]	Case study	Dubai	UAE Health System	Review of the necessary changes in the healthcare system in Dubai to accommodate population growth and burden of disease
Vetter, 2012 [15]	Case study	Abu Dhabi	Healthcare Financing	Strong regulatory focus on cost containment
			UAE Health System	Many changes since 2006, in particular introduction of mandatory insurance and the establishment of a regulator

### The UAE health system

Ten of the included papers discussed the UAE health system. Improving the quality of healthcare as well as the actual health outcomes for its citizens has been a key strategic goal of the UAE government since its formation in 1971. Dubai and Abu Dhabi have their own health authorities for licensing, regulation and quality assurance. The Federal Ministry of Health (MOH) fulfils these functions in the other five emirates. In addition the MOH carries out certain high level functions for all Emirates [12].

In both Dubai and Abu Dhabi around 70% of outpatient visits are made to private healthcare facilities while for inpatient activity in private facilities the proportion is 40% in Abu Dhabi and 60% in Dubai [13, 14]. In the remaining five Emirates, the Ministry of Health is both the regulator as well as the main provider of most healthcare services. According to the most recent data, in 2014, there were 36 government and 79 private hospitals in the UAE, an increase of 25% since 2009 [15].

In 2006 the government of Abu Dhabi embarked on a significant health system reform program with a clear focus on the redesign of the healthcare financing and regulatory system [8]. The regulatory function (the responsibility of Health Authority Abu Dhabi) was separated from service provision (the responsibility of the Abu Dhabi health service company, SEHA). Also the new system required all persons to have private health insurance and provides a centralized platform for automated claims processing and an improved level of accountability and transparency because of market regulation [16]. One study reported large differences in healthcare utilization rates between UAE nationals who, on average, used outpatient clinical services once per month compared to expatriates where usage rates were 3–4 times less [17].

In 2014, Dubai also began to introduce mandatory health insurance, with about one third of its residents currently estimated to be insured [18]. A recent review of the Dubai health system concluded that more effort should be made to move from curative to preventive services [19]. The same review also found that the current system of care encouraged excessive hospital utilization and recommended a reorientation towards outpatient, home based and day surgery services.

It has been reported that the rest of the UAE will follow soon with the introduction of mandatory private health insurance but a final date has not been set [20]. The MOH is considering introducing health insurance but has not yet done so. In the northern Emirates, the private sector is less well developed than in Dubai and Abu Dhabi and the quality and cost of services varies between these two Emirates and the remainder of the country [17].

Five of the studies examined the UAE health regulatory system with one highlighting the trend towards regulatory fragmentation as a serious challenge to the future of healthcare in the UAE [18]. A further study reported the lack of regulatory control and a lack of competition between insurance companies as the two main obstacles to achieving greater cost efficiency in the healthcare market [21]. Researchers who evaluated the regulatory system for healthcare professionals concluded that the UAE had made significant progress in developing and implementing best regulatory practice [12]. Other research on the regulation of healthcare services in Abu Dhabi concluded that several challenges remained to be addressed, in particular with respect to quality improvement [18]. Interestingly, Abu Dhabi’s healthcare regulator itself, HAAD, concluded in 2013 that “the current model of care in Abu Dhabi does not adequately support self-care or prevention and screening programs and diagnostic services are not integrated into care plans. Also, patients have undirected access to services and specialty care which leads to inappropriate use and, in turn, over-supply of services” [14].

### Population health

Five studies addressed this topic. The UAE population can be characterized as young and fast growing. The UAE population pyramid is remarkable in term its youthfulness and the high proportion of male expatriates [22]. Overall, the median age is 30 but amongst UAE nationals, who only account for approximately 11% of the population, 79% are aged less than 35 [3, 15]. Expatriates are typically of working age but despite this the majority are aged 35 or less. Population growth rate has also been remarkable. In 1950 the population was 70,000, in 1968 it was 180,000 but this has now grown to 9.16 million [23, 24]. Over the last 10 years the population has more than doubled, mainly due to large net in-migration of expatriates. Since the population of nationals is small, the contribution of the birth rate amongst nationals to overall population growth is also small. For example, between 2010 and 2014, the UAE population grew by over one million. However, during this four-year period, the national population increase by only 126,609 (births minus deaths). In other words, population growth amongst nationals contributed only 11.7% of total population growth. By comparison, natural growth amongst expatriates contributed 19% of total population growth and net in-migration contributed the remaining 70%. The great majority of the expatriate population in the UAE are male, young and originally from Asian countries. For example, it is estimated that approximately 2.6 million Indian nationals reside in the UAE [25]. The total fertility rate (average number of children that would be born to a woman over her lifetime) decreased from 4.4 in 1990 to 2.4 in 2010 [26]. During the same period, the average life expectancy improved from 72 years to 77 years [27]. The unique characteristics of the UAE population should play a major role in the development and implementation of health strategies and policies. Clearly child and maternal health services, youth services, health promotion and preventative services and occupational health services should be priorities [17]. A recurring theme from the studies that we reviewed is the need to improve health data collection and reporting [17, 22]. For example, birth and death data are reported but not by nationality, making it difficult to determine what, if any, specific, targeted strategies are required. In summary, the demographic transition in the UAE is one characterized by declining birth and death rates which with high net in-migration has resulted in significant population growth [28]. There has been a second health transition in the UAE in recent decades, an epidemiological transition, characterized by a decline in communicable diseases and a rise in non-communicable or chronic diseases, such as heart disease, diabetes and cancer [28]. This is described in the following section.

### The burden of disease

Eight studies discussed UAE mortality, morbidity and risk factors. As mentioned earlier, the UAE Government has set itself a number of challenging targets through its Vision 2021 strategy [5]. Of particular relevance are the targets to reduce the number of deaths (per 100,000 population) from cardiovascular disease from 297 to 158. Other targets relate to a reduction in the number of adults with diabetes (from 19 to 16%), a reduction of obesity amongst children (from 13 to 12%) and an increase in the healthy life expectancy (from 67 to 73 years). Since its independence in 1971, the UAE has made significant progress with increased life expectancy and lower maternal and infant mortality rates [29]. However, despite these achievements, the UAE faces a number of challenges including rising rates of non-communicable diseases such as diabetes, cardiovascular diseases and cancer [26].

The UAE has made progress with the control and prevention of communicable diseases, through a strong focus on immunization, surveillance, mandatory reporting and effective treatment [26]. The mandatory screening of all expatriate workers linked to the visa application and renewal process has also had an effect [30]. The national neonatal screening program for new born babies has been successful with an increased uptake from 50% in 1998 to 95% in 2010 resulting in early detection, treatment and follow up [31]. The WHO currently estimates that world-wide around 67% of all deaths are now attributable to non-communicable diseases, with the leading causes of death reported as cardiovascular diseases, injury and cancer [32]. This is also the situation in UAE, where mortality from non-communicable diseases (NCDs) among those aged 60 years or younger is amongst the highest in the world. The leading causes of premature deaths in the UAE are road injury, cardiovascular disease and respiratory illnesses [17]. In the studies that we reviewed, authors identified the determinants of this health loss as unhealthy lifestyles (physical inactivity, high caloric intake) and a lack of health system focus on prevention, chronic disease management, early stage interventions and inadequate treatment options for NCDs and their complications. As solutions, these authors proposed further research, the establishment of reliable surveillance and monitoring programs and improved training and education for healthcare professionals [26, 31, 33].

We found five studies that described interventions to address the UAE burden of NCDs. One such intervention is the Abu Dhabi Weqaya program that aims to screen adults for cardiovascular disease risk factors followed by targeted follow up, treatment and secondary prevention [34]. Weqaya has confirmed a high prevalence of cardiovascular disease risk factors amongst the adult population. Following the successful implementation of screening in a small, high-risk population using newly agreed UAE screening guidelines other researchers have recommended a national diabetes screening program [35]. In the review we found a number of studies that reviewed the direct and indirect economic burden of selected diseases, including asthma and diabetes [33, 36]. The economic burden of asthma was estimated at US$ 29 million in Abu Dhabi and US$ 24 million in Dubai, an annual per capita cost of around US$ 200, about half the cost compared to European or North American benchmarks. One of the most cited articles in the review assessed the direct medical costs of diabetes care, the annual cost of diabetes without complications was US$ 1605, similar to the costs in most western countries but the treatment costs of diabetes mellitus with complications was up to 9.4 times higher [33]. The authors of these papers that reviewed the economic costs of high burden diseases typically recommended improvements in management including nationwide early screening and rapid implementation of best-practice clinical guidelines as a means to improve outcomes while controlling costs.

### Healthcare financing

**Fig. 3:**
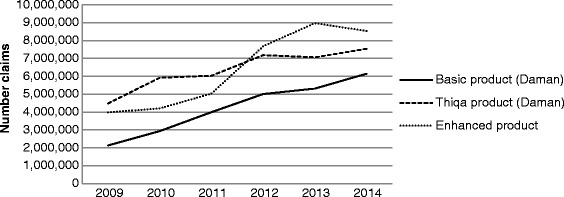
Health insurance claims by type of insurance scheme, Abu Dhabi, 2009–2014

Seven studies discussed healthcare financing. Recently published WHO data indicates that in the UAE over the last 12 years, total expenditure on health as percentage of gross domestic product (GDP) has increased by over 36% (from 2.2% of GDP in 2000 to 3.0% in 2012) [3]. In absolute terms, the UAE’s GDP rose from US$104.3 billion in 2000 to US$372.3 billion 2012, meaning that health spending grew from US$2.3 billion to US$ 11.2 billion. More recent reports show a further increase to US$13.6 billion in 2014 with an expected budget of US$25.7 billion by 2024 [37]. In Abu Dhabi, mandatory health insurance for all nationals and expatriates has been the major driver of its healthcare reform since 2006 [38]. There are three different insurance schemes: two for expatriates (Basic and Enhanced) and one for UAE nationals (Thiqa). In 2011 there were 15.3 million insurance claims with an average cost per claim of $105 giving a total insurance bill of US$ 1.6 billion. This had grown to over 22 million claims and US$2.9 billion by 2014 (Additional file 1) [14]. Even though there has been a steady rise in the number of claims (Fig. 3) as well as the overall cost, some researchers have argued that this is appropriate because universal health insurance cover and transparent, standardized payment rules and regulation allow for better control of cost, ensure that health needs are met and offer patients the freedom to choose provider [8, 16]. However, other researchers have concluded that increasing claims and costs signal the need for further changes to ensure long-term financial sustainability [38]. The WHO and other sources estimate that the UAE government spent almost a quarter of its total healthcare expenditure in 2010 to send its citizens abroad for medical care [16, 32]. Dubai Health Authority for example sponsored 2717 patients in 2014 for treatment abroad, an increase of almost 2000 in 10 years (Table 2) [13]. While in 2013, Health Authority Abu Dhabi sponsored over 1400 patients [14]. Also there are other referral sources for UAE nationals who wish to be sent abroad for medical treatment, including the Ministry of Health, Ministry of Defense, Abu Dhabi National Oil Company (ADNOC) and other large companies [8].

**Table 2:** Funding of International Patient Care by Dubai Health Authority

Year	No. UAE Patients who received medical treatment outside UAE	Average cost per patient (US$)	Total cost (US$)
2004	808	40,436	32,672,262
2005	679	54,768	37,187,738
2006	863	57,221	49,381,471
2007	946	51,499	48,717,711
2008	850	75,204	63,923,706
2009	1073	59,128	63,444,414
2010	975	68,392	66,682,561
2011	1428	57,766	82,489,373
2012	1819	50,681	92,189,101
2013	2010	46,921	94,311,172
2014	2717	44,142	119,932,970

Source: Dubai Health Authority’s Annual Reports [43]

At the same time, the UAE is working to attract medical tourists to its healthcare facilities, in particular its highly specialized hospitals. For example in 2012, Dubai attracted over 500,000 medical tourists, a figure that is expected to grow annually by 10–15% [39]. In the UAE the level of out-of-pocket (OOP) healthcare expenses is relatively low in comparison to other countries in the region and the rest of the world. At 20% the OOP is just above the OECD average of 17% indicating a reasonable level of financial protection [40]. A number of studies have commented on the low levels (ranging from 4 to 15%) of generic prescribing and the high use of branded pharmaceuticals with the inevitable implications for increasing costs [41, 42]. In the UAE, data on health care spending is not yet available in a standardized format. The claims based data for Abu Dhabi shown in Table 2 contains only the reimbursement cost not the actual cost. Denials, co-payments and sole payments are not included. Also cost estimates typically exclude capital expenditure, funding provided through other government institutions such as the Ministry of Defense and ADNOC and cash payments. Table 3 gives a breakdown and estimate of the healthcare expenditure in the UAE based on our findings from this review [19, 43].

**Table 3:** UAE total healthcare expenditure (Billion US$), by Emirate, 2014

	Healthcare Expenditure (Billion US$)
Abu Dhabi	4
Dubai	3.5
Northern Emirates	2.5
International Patient Care	3.6
Total	13.6

Source: Dubai Health Authority, Health Authority Abu Dhabi, World Health Organization, US UAE Business Council

### Healthcare infrastructure and workforce

Five studies addressed this topic. Hospital bed and physician and nurse numbers have increased in the past decade generally keeping pace with the growth of the population (Table 4). The total number of hospital beds has more than doubled and there has almost been a five-fold increase in the number of nurses and physicians [15]. A number of case studies have reviewed the current demand and supply and made recommendations for future configuration and capacity. Few of these studies reported that additional increases in hospital beds and staff numbers were justified [17, 37, 44]. However, Health Authority Abu Dhabi estimated that a further 4800 physicians and 13,000 nurses would be required for Abu Dhabi alone to meet the projected 2022 demand [14]. The goal for the UAE is to bring the level of nurses and physicians to a world class level, which means that the number of nurses need to be almost doubled and the number of physicians needs to increase by 20% [5].

**Table 4:** UAE healthcare infrastructure, by category, Government and private, 2005–2014

	2005	2010	2014
Government^*^			
Hospitals	26	34	36
Beds	4273	7029	7493
Physicians	2105	5031	6504
Nurses	6132	10,875	16,547
Private			
Hospitals	37	58	79
Beds	1546	2556	4164
Physicians	1143	7866	10,165
Nurses	1866	10,611	16,882
Total			
Hospitals	63	92	115
Beds	5819	9585	11,657
Physicians	3248	12,897	16,669
Nurses	7998	21,486	33,429

Source: UAE Federal Competitiveness and Statistics Authority

*Includes Ministry of Health, Ministry of Interior, Ministry of Defense, Abu Dhabi Health Authority, Dubai Health Authority and ADNOC [16]

Despite these reported shortfalls in capacity and resources, the authors were unable to find any studies that analyzed the potential effects of the reported lack of manpower and hospital beds. On the contrary, a number of studies, as well as a report from the Abu Dhabi regulatory authority described potential oversupply in certain areas [14, 17]. Another challenge is the high rate of turnover of clinical staff, with one report estimating that around 15% of physicians and 13% of nurses left their positions in the UAE in 2012 alone [45].

### The impact of reforms: Quality

Only three of the studies focused on the impact of health system reforms. Although a number of researchers have commented that it is too early to say whether the UAE health system reforms that have been in place over the past 10 years have achieved the desired outcomes, there is evidence of a positive trend [8, 17]. A recent study in a large hospital in Abu Dhabi found a decrease in reported clinically significant adverse events in one department (pediatrics) over a 4 year period [46]. This decrease coincided with the reform of its residency training program, leading to the researcher’s conclusion that “it is quite likely that our residents are providing better patient care”. In Abu Dhabi, a study into perceptions and attitudes towards medical research amongst focus group participants noted that the UAE has one of the best healthcare systems in the region [47]. The UAE has also witnessed a significant growth in *Joint Commission International* (JCI) accreditation [48]. JCI accreditation has become increasingly important in the UAE, where a growing number of providers have become accredited (Table 5) [49]. It is estimated that currently 47% of healthcare facilities are accredited and the UAE government’s ambition is to achieve 100% accreditation by 2021 [5]. In our review we found few studies that reported quality and outcomes of care. However, in one study in Dubai that reviewed the quality of care for diabetic patients, using a standardized assessment, the researchers found a number of differences when compared to the US benchmark and recommended a nationwide benchmarking program [50]. Another study found that while a private hospital maintained its performance following JCI accreditation, accreditation did not contribute to an overall, sustained improvement [51]. Finally, in our review, we found that studies that examined patient satisfaction generally reported consistently high levels compared to other countries [8, 17].

**Table 5:** Joint Commission International accredited facilities, UAE, 2007–2015

Year	No of healthcare facilities with JCI accreditation
2007	14
2008	18
2009	33
2010	42
2011	49
2012	55
2013	82
2014	102
2015	116

Source: Joint Commission International

## Discussion

This review has highlighted the ambition and commitment of the UAE to build a world class health system and has catalogued the major reforms that have been implemented in the past decade to achieve this. The paucity and limited scope of the studies means that it is not possible to conclude whether the reforms are working although patient satisfaction with services appears high and there are some isolated examples of quality improvement.

The UAE health system is not a single system, rather there are several systems and of these the three main systems are operated by the health authorities of Abu Dhabi and Dubai and the Ministry of Health (MOH). These systems have expanded in the past 10 years in line with the growth of the population and increases in national income and have been subjected to major reforms aimed at improving public health and quality while keeping costs at sustainable levels, thereby achieving a world class health service. The main element of the reforms have been a move to mandatory private health insurance for all citizens and expatriates, the development of the private sector to deliver services and the separation of planning and regulatory responsibilities from provider functions. These reforms have moved at different speeds, being most complete in Abu Dhabi, in the development phase in Dubai and just commencing in the MOH. This patchy implementation has highlighted variations in access, affordability and quality across the Emirates. Amongst researchers and commentators opinion is divided on whether the reforms have been successful. Few, if any, studies have prospectively set out to define and measure outcomes and while some researchers have expressed optimism others have been more critical. The relevance of the reforms for public health and their impact on the determinants of chronic diseases have been questioned with some researchers citing market failure and oversupply.

The UAE has a rapidly growing population with a unique age and sex distribution. There is an unusually high proportion of young people and expatriates of working age, small numbers of older persons and rapid year on year growth due to high net in-migration. It might be expected that the unique characteristics of the population would be a major factor to be considered when planning and implementing health services but there is little published research to support this. While child and maternal health services are well developed, there is little published evidence of needs analysis in the areas of youth services, health promotion, preventative services and occupational health services. Also health data is not collected and reported in a way that allows the health needs of these population sub-groups to be defined.

The UAE has passed through the epidemiological transition with impressive reductions in health loss from infections and neonatal and nutritional disorders but an increasing burden of non-communicable disease (NCD) notably cardiovascular disease (CVD), diabetes and road injury. The lifestyle risk factors for these diseases (obesity, low physical activity) are at high levels. From our review there is evidence of high level commitment to addressing these issues. The Abu Dhabi *Weqaya* program set out to identify and manage individual CVD risk factors but after the initial report describing the program and presenting baseline data there have been no updates on outcomes, effectiveness or recommendations to extend the program to the whole UAE adult population. There is good evidence for the considerable cost burden that NCDs place on health budgets and bench-marking has shown that the situation in the UAE is comparable to that in other high income countries. However there is also evidence that in the management of NCDs international best practice is not always followed.

Total expenditure on health has increased both in absolute terms and as a percentage of national income. As in all health systems these increases can be explained on the basis of population growth, aging of the population, advances in technology and price inflation. In the UAE, the increases may also be justified if there was previously unmet need that is now being met. However in our review we found researchers who suspected over-use, waste and fraud and who questioned whether the increases in activity and cost were sustainable or whether further reforms were required. In the review, a recurring theme was the need to economize on drug costs by encouraging greater use of generic products. In our review we were surprised that, given the excellence of the UAE health system, substantial numbers of patients are funded to have medical treatment abroad at substantial cost. This is all the more noteworthy because the UAE health system is highly successful at attracting incoming medical tourists. The reasons for this curious state of affairs was not explored in depth but if the UAE’s ambition to have a world class health system is fully achieved then funding patients to receive routine treatment abroad would seem to be improvident. In the review we found discussion of the percentage of total health expenditure that is contributed by out-of-pocket (OOP) expenses, a widely used metric to indicate financial security. In the UAE, the OOP percentage is comparable to that seen in other countries with well-developed progressive health systems. This might appear surprising given the high levels of disposable income enjoyed by many UAE citizens and expatriates. However, once again our review highlighted the need to improve the quality of data collection and reporting and to make allowance for the fact that the UAE population is very heterogeneous.

In this review, we found that a normative approach was typically adopted to plan and predict future capacity both for hospital bed numbers and numbers of doctors, nurses and other healthcare staff. The norms or benchmarks that are used are those from North America and Europe. It is not clear if there is shortage or oversupply or what, if any, are the consequences of this. What is clear from published evidence is the high staff turnover and poor retention rates.

From our review, it is not possible to say if the UAE health systems reforms are working. Some researchers have concluded that it is too early to expect to see any effect but mostly the research in this area has not focused specifically on this question. We found isolated reports of initiatives that have improved quality. UAE national policy is that all hospitals should be JCI accredited and good progress is being made towards this target. Again, there are a few reports of the beneficial effects of accreditation but this is an area that is poorly researched. We found isolated examples of where services or programs had been audited against international best practice benchmarks with mixed findings. Where researchers commented on patient satisfaction with services this was usually high.

Despite the increased focus on healthcare reforms in many countries, it remains a concept lacking a clear definition [9]. According to one definition, health system reform can be described as a “significant purposive effort to improve the performance of the health care system” [52]. With respect to the impact of reforms, several authors have cautioned against simplistic, cause-and-effect logic because of the complexities involved in overseeing and providing healthcare with multiple, demanding stakeholders, competing political priorities and high expectations [53, 54]. However, despite this caution, over the last three decades, global institutions such as the World Health Organization and the World Bank have stimulated national government to reform their health systems, with notable results [55].. For example, governments of developing countries, such as Brazil, Russia, India, China and South Africa have committed themselves to radical reform programs with the goal of achieving universal health coverage and China in particular has made significant progress in ensuring that its population has access to healthcare [56, 57]. Similarly, the Affordable Care Act in the US has resulted in an impressive decrease in the percentage of uninsured adults [58].. Specifically to the Middle East and North Africa region, researchers have commented on the increased focus on building or reforming health insurance systems as a popular method of reform [59].

This is the most complete summary, to date, of the evidence available on the progress and outcomes of health systems reform in the United Arab Emirates. Our study is not without limitations. We found a limited number of studies that addressed UAE health system reform and of those that did most lacked robust methodology and failed to focus on the outcomes of reform. Although our search strategy was broad and included both published and unpublished sources to minimize publication bias it is possible that papers meeting our inclusion criteria were missed and therefore, not included in the review. Nevertheless, the review provides a stock-take or baseline from which future researchers can plan and develop their research questions. We have identified some important gaps in knowledge that may inform future research.

## Conclusion

The UAE government is committed to build a world class health system to improve the quality of healthcare health outcomes for its population. To achieve this it has implemented extensive health system reforms in the past 10 years including the introduction of mandatory private health insurance, the development of the private sector and the separation of planning and regulatory responsibilities from provider functions. From the existing research literature it is not possible to conclude whether the reforms are working although there are some positive indications including high patient satisfaction, increasing coverage of JCI accreditation and isolated examples of quality improvement. We recommend that research should continue in this area but that research questions should be more clearly defined focusing whenever possible on outcomes rather than processes. In addition there is need for better quality data collection and reporting to allow the health needs and outcomes of specific population sub-groups to be defined. Finally there is scope to align services and program more closely with international best practice and to benchmark UAE performance with that of similar highly developed, progressive health systems from around the world.

## Additional file

Additional file 1: Table. Insurance costs, Abu Dhabi, 2011–2014 (DOCX 15 kb)

## Abbreviations

ADNOC: Abu Dhabi National Oil Company
CVD: Cardiovascular disease (CVD)
DHA: Dubai Health Authority (DHA)
EMRO: Regional Office for the Eastern Mediterranean Region (of WHO)
GDP: Gross domestic product
HAAD: Health Authority Abu Dhabi
JCI: Joint Commission International
KPI: Key performance indicator
MMR: Maternal mortality rate
MOH: Federal Ministry of Health
NCD: Non-communicable disease
OECD: Organisation for Economic Co-operation and Development
OOP: Out-of-pocket (healthcare expenses)
PRISMA: Preferred Reporting Items for Systematic Reviews and Meta-Analyses
SEHA: Abu Dhabi Health Services Company
UAE: United Arab Emirates
WHO: World Health Organization
