# Supplementary material for: Progress and outcomes of health systems reform in the United Arab Emirates: a systematic review
Source: BMC Health Serv Res. 2017 Sep 20;17:672. doi: 10.1186/s12913-017-2597-1 (PMC5607589; doi:10.1186/s12913-017-2597-1)
Supplement: Additional file 1: — Table. Insurance costs, Abu Dhabi, 2011–2014 (DOCX 15 kb) [file 12913_2017_2597_MOESM1_ESM.docx]

Table. Insurance costs, Abu Dhabi, 2011-2014

| **Cost of average insurance claim, 2011 (US$)** | | | | | | | |
| --- | --- | --- | --- | --- | --- | --- | --- |
|  | Inpatient Claims | | | Outpatient Claims | | |  |
|  | Costs | No. Claims | Total costs | Costs | No. Claims | Total costs |  |
| Basic product (Daman) | 2,396 | 35,000 | 83,847,411 | 41 | 3,952,000 | 163,679,564 |  |
| Thiqa product (Daman) | 3,468 | 54,000 | 187,263,760 | 99 | 5,976,000 | 589,458,311 |  |
| Enhanced product | 2,546 | 34,000 | 86,565,668 | 93 | 4,992,000 | 466,554,768 |  |
| Total costs |  |  | 0.4 billion |  |  | 1.2 billion | 1.6 billion |
| **Cost of average insurance claim, 2012 (in USD)** | | | | | | | |
|  | Inpatient Claims | | | Outpatient Claims | | |  |
|  | Costs | No. Claims | Total costs | Costs | No. Claims | Total costs |  |
| Basic product (Daman) | 2,416 | 42,000 | 101,486,649 | 40 | 4,964,000 | 198,830,518 |  |
| Thiqa product (Daman) | 4,053 | 72,000 | 291,805,995 | 115 | 7,100,000 | 818,337,875 |  |
| Enhanced product | 2,775 | 52,000 | 144,310,627 | 87 | 7,620,000 | 666,490,463 |  |
| Total costs |  |  | 0.5 billion |  |  | 1.7 billion | 2.2 billion |
| **Cost of average insurance claim, 2013 (in USD)** | | | | | | | |
|  | Inpatient Claims | | | Outpatient Claims | | |  |
|  | Costs | No. Claims | Total costs | Costs | No. Claims | Total costs |  |
| Basic product (Daman) | 2,475 | 44,000 | 108,885,014 | 47 | 5,260,000 | 249,384,196 |  |
| Thiqa product (Daman) | 3,934 | 88,000 | 346,197,275 | 140 | 6,968,000 | 974,001,090 |  |
| Enhanced product | 2,768 | 66,000 | 182,659,946 | 92 | 8,841,000 | 816,648,229 |  |
| Total costs |  |  | 0.7 billion |  |  | 2 billion | 2.7 billion |
| **Cost of average insurance claim, 2014 (in USD)** | | | | | | | |
|  | **Inpatient Claims** | | | **Outpatient Claims** | | |  |
|  | Costs | No. claims | Total costs | Costs | No. claims | Total costs |  |
| Basic product (Daman) |  | 45,000 |  |  | 6,087,000 |  |  |
| Thiqa product (Daman) |  | 60,000 |  |  | 7,468,000 |  |  |
| Enhanced product |  | 76,000 |  |  | 8,454,000 |  |  |
| Total costs | 3,648 | 181,000 | 0.7 billion | 98 | 22 million | 2.2 billion | 2.9 billion |

Source: Health Authority Abu Dhabi [12]
